# Italian Guidelines for the Management of Non-Functioning Benign and Locally Symptomatic Thyroid Nodules

**DOI:** 10.2174/1871530323666230201104112

**Published:** 2023-04-19

**Authors:** Enrico Papini, Anna Crescenzi, Annamaria D’Amore, Maurilio Deandrea, Anna De Benedictis, Andrea Frasoldati, Roberto Garberoglio, Rinaldo Guglielmi, Celestino Pio Lombardi, Giovanni Mauri, Rosa Elisa Miceli, Soraya Puglisi, Teresa Rago, Domenico Salvatore, Vincenzo Triggiani, Dominique Van Doorne, Zuzana Mitrova, Rosella Saulle, Simona Vecchi, Michele Basile, Alessandro Scoppola, Agostino Paoletta, Agnese Persichetti, Irene Samperi, Renato Cozzi, Franco Grimaldi, Marco Boniardi, Angelo Camaioni, Rossella Elisei, Edoardo Guastamacchia, Giulio Nati, Tommaso Novo, Massimo Salvatori, Stefano Spiezia, Gianfranco Vallone, Michele Zini, Roberto Attanasio

**Affiliations:** 1 Department of Endocrine and Metabolic Diseases, Ospedale Regina Apostolorum, Albano Laziale, Rome, Italy;; 2 Department of Endocrine Organs and Neuromuscolar Pathology, Università Campus Bio-Medico di Roma, Rome, Italy;; 3 Endocrine Surgery Division, Agostino Gemelli School of Medicine, University Foundation Polyclinic, Rome, Italy;; 4 Endocrinology and Center for Thyroid Diseases, Ospedale Mauriziano “Umberto I”, Turin, Italy;; 5 Quality Management - Clinical Direction, Fondazione Policlinico Universitario Campus Bio-Medico, Rome, Italy;; 6 Struttura Complessa di Endocrinologia, Arcispedale S. Maria Nuova, IRCCS, Reggio Emilia, Italy;; 7 Freelancer at Thyroid Multidisciplinary Center at Humanitas Cellin, Turin, Italy;; 8 Interventional Radiology, IRCCS European Institute of Oncology, Milan, Italy;; 9 Private Practice, Rome, Italy;; 10 Department of Clinical and Biological Sciences, Internal Medicine, AOU San Luigi di Orbassano, University of Turin, Turin, Italy;; 11 Department of Clinical and Experimental Medicine, University of Pisa, Pisa, Italy;; 12 Department of Public Health, University Federico II, Naples, Italy;; 13 Interdisciplinary Department of Medicine-Section of Internal Medicine, Geriatrics, Endocrinology and Rare Diseases, University of Bari, Bari, Italy;; 14 Associazione Medici Endocrinologi, Relationship with Patients’ Associations, RomeItaly;; 15 Department of Epidemiology, Lazio Region Health Service, Rome, Italy;; 16 High School of Economy and Management of Health Systems, Catholic University of Sacred Heart, Rome, Italy;; 17 Department of Endocrinology, Ospedale Santo Spirito, Rome, Italy;; 18 Department of Endocrinology, ULSS6 Euganea, Padova, Italy;; 19 Department of Firefighters, Public Rescue and Civil Defense, Ministry of Interior, Rome, Italy;; 20 Department of Endocrinology, ASL Novara, Novara, Italy;; 21 President of Associazione Medici Endocrinologi, Milan, Italy;; 22 Past-president of Associazione Medici Endocrinologi, Udine, Italy;; 23 General Oncologic and Mini-invasive Surgery Department, ASST Grande Ospedale Metropolitano Niguarda, Milan, Italy;; 24 Otolaryngology Department, San Giovanni-Addolorata Hospital, Rome, Italy;; 25 ASL Roma, Rome, Italy;; 26 Department of Endocrinology, Santa Maria Nuova Hospital, Turin, Italy;; 27 Nuclear Medicine Unit, Fondazione Policlinico Universitario A. Gemelli IRCCS and Department of Radiological and Hematological Sciences, Catholic University of Sacred Heart, Rome, Italy;; 28 Department of Endocrine and Ultrasound-Guided Surgery, Ospedale del Mare, Naples, Italy;; 29 Department of Radiology, Federico II University Hospital, Naples, Italy;; 30 AME Scientific Committee, Milan, Italy

**Keywords:** Thyroid nodule, thyroidectomy, hemi-thyroidectomy, ablation, thermo-ablation, radiofrequency, laser, microwave, HIFU, ultrasound, ethanol injection

## Abstract

***Aim*:** This guideline (GL) is aimed at providing a reference for the management of non-functioning, benign thyroid nodules causing local symptoms in adults outside of pregnancy.

***Methods*:** This GL has been developed following the methods described in the Manual of the National Guideline System. For each question, the panel appointed by Associazione Medici Endocrinology (AME) identified potentially relevant outcomes, which were then rated for their impact on therapeutic choices. Only outcomes classified as “critical” and “important” were considered in the systematic review of evidence and only those classified as “critical” were considered in the formulation of recommendations.

***Results*:** The present GL contains recommendations about the respective roles of surgery and minimally invasive treatments for the management of benign symptomatic thyroid nodules. We suggest hemithyroidectomy plus isthmectomy as the first-choice surgical treatment, provided that clinically significant disease is not present in the contralateral thyroid lobe. Total thyroidectomy should be considered for patients with clinically significant disease in the contralateral thyroid lobe. We suggest considering thermo-ablation as an alternative option to surgery for patients with a symptomatic, solid, benign, single, or dominant thyroid nodule. These recommendations apply to outpatients, either in primary care or when referred to specialists.

***Conclusion*:** The present GL is directed to endocrinologists, surgeons, and interventional radiologists working in hospitals, in territorial services, or private practice, general practitioners, and patients. The available data suggest that the implementation of this GL recommendations will result in the progressive reduction of surgical procedures for benign thyroid nodular disease, with a decreased number of admissions to surgical departments for non-malignant conditions and more rapid access to patients with thyroid cancer. Importantly, a reduction of indirect costs due to long-term replacement therapy and the management of surgical complications may also be speculated.

## 1 INTRODUCTION

Thyroid nodules are detected in 5-7% of the adult population at physical examination [1] and in up to 60% of adult females as an incidental finding during imaging procedures [2, 3]. Most nodules are benign, but 5-15% may be malignant, with a risk level depending on sex, age, and medical history [2, 3]. Fine needle aspiration (FNA) is a reliable procedure to stratify the risk of malignancy [2, 3] and no therapy is required for cytologically benign, non-hyperfunctioning thyroid nodules if they are asymptomatic [4]. However, a low but not negligible fraction of these nodules progressively grows until local pressure symptoms develop [4].

The diagnostic workup and the therapeutic management of this frequent clinical problem drains considerable health resources, while patients’ quality of life (QoL) may be negatively affected by aggressive treatment modalities. Data from the USA demonstrate a progressive increase in thyroid surgery (+39% from 1996 to 2006) [5] and in total thyroidectomies for benign disease (from 17.6% during 1993-1997 to 39.4% during 2003-2007) [6]. European data are aligned: 60% of thyroid surgeries are performed in France for benign nodular thyroid disease [7] and only 30% of thyroid surgeries for benign diseases in Germany are due to hyperthyroidism or local pressure symptoms [8].

An appropriate work-up for nodular thyroid disease, aimed at decreasing unwarranted surgery, should follow the sequential steps listed below [1-3, 9-12].

1. History: pay attention to family history of thyroid surgery, thyroid tumors, genetic syndromes associated with thyroid neoplasms, and personal history of external radiation or nuclear fall-out.
2. Physical examination: pay attention to the anterior and lateral neck for lymphadenopathy.
3. Laboratory: first assay thyrotropin [TSH) to rule out alterations of thyroid function. If they are present, free thyroid hormone levels should be determined. If TSH is low, perform a thyroid scintiscan to disclose regional hyperfunction. Hot nodules are generally benign and do not require cytologic examination. If TSH is high, anti-thyroid antibodies should be evaluated. Normal serum calcitonin reliably rules out medullary thyroid carcinoma.
4. Neck ultrasound (US): examination should be performed by a skilled operator with a high-frequency linear probe. Malignancy risk classification should be applied to the nodule according to EU-TIRADS system [1-3, 9, 10, 13].
5. US-guided FNA: thyroid sampling should be performed according to an algorithm integrating clinical suspicion, nodule size, and US risk classification [9, 10, 13]. Use a validated classification for cytology reporting [14], mainly the 2014 Italian Consensus for the Classification and Reporting of Thyroid Cytology [15]. In several major series, malignancy risk is lower than 3% for the TIR2 category.

The standard treatment for nodular thyroid disease that causes clinical symptoms is surgery [2]. Selective resection of the nodule is no more employed, due to the high rate of peri- and post-operative complications and recurrence. The resection of the affected lobe and isthmus is currently suggested for benign monolateral nodules and also for differentiated thyroid carcinomas (DTC) staged as clinically pT1 and pT2 [2, 11]. This approach prevents the need for complete replacement therapy [16]. Conversely, total thyroidectomy remains the first choice for bilateral multinodular goiter, Graves’ disease, and advanced DTC. Importantly, high-volume thyroid surgeons achieve better outcomes with a lower rate of complications [17-19]. A suggested cut-off is 50 thyroidectomies yearly per surgeon or 100 thyroidectomies per center [20].

In the last decades, image-guided non-surgical procedures (MIT) have become available for the management of benign thyroid nodules, aiming to relieve of local pressure symptoms [21]. MIT includes chemical ablation with ethanol injection [PEI] [22, 23] and thermal ablation with laser [LTA] [24], radiofrequency [RFA] [25-27], microwaves [MWA] [28, 29], or high intensity focused ultrasounds (HIFU) [30]. The long-term follow-up of these procedures is still limited (up to 5 years in most series) and in 10-15% of the cases, a partial regrowth of the nodule occurs, warranting further treatment [21].

TSH-suppressive treatment with levo-thyroxine (LT4) [31-33] and radioiodine ablation [34] are no more employed in this setting due to low efficacy and potential side effects.

Due to the high prevalence of benign nodular disease, the management algorithm should be based on the risk-benefit and cost-efficacy ratios of the different procedures as well as on their accessibility. Physicians should advice patients about the therapeutic options, and inform them about the risks and benefits of the available procedures and of their impact on QoL to allow a shared decision with patients.

The aim of this guideline (GL) is to answer the question: What is the efficacy of hemithyroidectomy plus thyroid isthmus resection *vs*. total thyroidectomy *vs*. ablative procedures, *vs*. other non-invasive treatments, *vs*. no treatment for patients with benign symptomatic thyroid nodules?

## 2 METHODS

This GL was developed according to the Methodological manual for the production of clinical practice GLs developed by the National Center for the Clinical Excellence, Quality and Safety of Care of the Italian National Institute of Health (http://www.snlg-iss.it). Appendix **1** details the names and roles of all the people involved in the GL development team.

### 2.1 Clinical Questions

The recommendations are the answers to a clinical question, formulated by the panel using the Population-Intervention-Comparison-Outcome (PICO) framework (Appendix **2**).

### 2.2 Selection of Outcomes

For each question, the panel identified potentially relevant outcomes, which were then rated for their impact on therapeutic choices using a 9-point scale, namely:

➢ 1–3 points: outcomes of limited relevance

➢ 4–6 points: important, but not critical outcomes

➢ 7–9 points: critical outcomes.

Only outcomes classified as “critical” and “important” were considered in the systematic review of evidence and only those classified as “critical” were considered in the formulation of recommendations [35].

### 2.3 Literature Review and Assessment of the Quality of Evidence

A systematic search for each question was performed on the following databases: Cochrane Library, MEDLINE, Embase, Web of Science, and CINAHL (from inception to October 2020).

Specific search strategies were used for each database, as specified in each section of Appendix **3**. No time or language limits were imposed for all the searches. References of retrieved items were searched for further studies meeting inclusion criteria.

A systematic review was performed through the following steps:

1. Selection of the eligible studies obtained with the initial search, based on title and abstract, for retrieval as full text.
2. Identification among retrieved full-text items of relevant studies, based on a priori inclusion and exclusion criteria.
3. Assessment of potential bias using validated instruments (Cochrane criteria for RCT – [36] - and checklist Newcastle-Ottawa Scale for observational studies – [37]).
4. Extraction of main characteristics of the selected studies (enrolled population, considered outcomes, results), summarized in tables.
5. Quantitative synthesis for each outcome, calculating odds ratio (OR) for categorical outcomes and weighted mean difference (WMD) for continuous variables with 95% confidence intervals (CI). Quantitative meta-analysis was performed with RevMan 5.4 using fixed effects models.
6. Assessment of heterogeneity (I^2^) by the I^2^ statistic stating the percentage of variability in effects esteem due to heterogeneity rather than to chance.
7. The overall quality and strength of available evidence for outcomes selected by the panel were rated using the GRADE criteria.
8. Synthesis of results, using the GRADEPro Guideline Development tool (https://gradepro.org), with the frameworks EtD, which summarize results of systematic reviews for problem priority, desired and undesired effects of treatments, the strength of available evidence, values and preferences of stakeholders, economic resources needed, equity, acceptability, and feasibility of interventions.

### 2.4 Pharmacoeconomic Studies

The economic evaluation was performed by a pharmacoeconomist with specific expertise (MB).

Since official data about costs of surgery and thermoablation for thyroid nodules were not always available, we performed a survey among panel members from different disciplines and regions that were representative of the Italian health system setting, looking for specific drivers that contribute to the total cost of each procedure (either surgery or thermoablation). Specifically for each procedure, we investigated the duration, type, and dosage of employed drugs, type and quantities of disposable materials, number and time of involvement of each operator, and percentage of patients requiring a caregiver during and after the procedure (indirect costs).

We calculated the mean value for each parameter to allow their use in the different regional settings under Italian National Health Service (NHS).

The Activity based costing (ABC) analysis is a useful tool to calculate resource employment and evaluation of the gross cost of procedures. ABC follows three steps:

1. Resource identification using a specific survey among the interdisciplinary panelists. The resources required to provide the investigated procedures were detailed to quantify each component (time of operators’ activities, materials, drug dosage, technical resources, *etc*.).
2. Cost determination by consultation with scientific literature and specific databases (such as price lists) [38-43].
3. Valorization of results: the data obtained during the previous steps were combined to define the aggregate value of each action and the whole process [44].

The economic analysis evaluated the four large resource categories employed in the procedure under investigation:

➢ The direct cost paid by NHS for drugs.

➢ The direct cost paid by NHS for disposable materials.

➢ The direct cost paid by NHS for the working time of operators and the use of structures.

➢ Indirect costs sustained by patients and caregivers.

To assess the costs driven by potential complications, we evaluated the rate of occurrence for each complication of the various procedures and we expressed the relative cost as the corresponding fraction. Namely, if the cost of a specific complication was € 5000, including all the drivers (employed drugs, hospital stay, and loss of productivity), and if the complication is reported to occur in 1% of patients, the sum of € 50 was added to the total cost of the procedure.

### 2.5 Development of Recommendations

The GL panel examined and discussed each clinical question: the EtD frameworks, the tables of evidence, and the summaries of results (forest plots of meta-analyses). The GL panel formulated recommendations (which were rated as strong or weak) based on the priority of the problems, benefits, and harms of the options, strengths of evidence, values and preferences, use of resources, feasibility, acceptability, and equity of the procedure.

Disagreements were settled through collective discussion.

If evidence was not available or it was inappropriate for a formal rating of the quality of evidence, the GL panel developed indications for good clinical practice, which should be considered complementary to recommendations.

### 2.6 External Review

The panel appointed a board of external reviewers with specific expertise in thyroid disease management. External reviewers received a draft version of the GL and submitted their comments to the panel, which included, after a dedicated discussion, the amendments to the GL document.

### 2.7 Value of Recommendations

Quality of evidence was rated as:

➢ High: highly reliable data whose confidence in estimated effects is very unlikely to be modified by further studies.

➢ Moderate: moderately reliable data whose confidence in estimated effects could be modified by further studies.

➢ Low: still limited and uncertain results which need further research for a reliable assessment of the positive and negative effects of the intervention.

➢ Very low: available data are not reliable and the estimates of effects should be considered with caution.

The strength of recommendations was rated as strong or weak.

A strong recommendation implies:

➢ For clinicians: the majority of patients should receive the recommended intervention.

➢ For patients: almost all properly informed patients should follow the recommendation whereas only a small fraction of them may choose different options.

➢ For the policy makers: the recommendation can be employed for planning the use of the available resources.

A weak recommendation implies:

➢ For clinicians: the final choice should include careful consideration of patients’ values and preferences.

➢ For patients: the majority of properly informed patients will follow the recommendation, but a minority of them may choose different options.

➢ For the policy makers: a discussion involving the stakeholders should be performed on the issue.

## 3 RESULTS

The PRISMA flow diagram for the selection of the studies is illustrated in Appendix **4**. Two randomized controlled trials (RCT) [45, 46] and 12 observational studies (nine retrospective *–* [47-55] and three prospective *–* [56-58]) met all the inclusion criteria. The methodological quality evaluation of selected studies is detailed in Appendix **4** and Appendix **5**, respectively.

No studies that compared thyroidectomy with TSH-suppressive or semi-suppressive treatment with LT4, iodine supplements, food integrators, radioiodine treatment, or clinical observation were retrieved.

### 3.1 Comparison between Hemithyroidectomy and Thyroidectomy

One RCT [45] and eight observational studies evaluated this topic [56, 58].

Major peri-procedural complications were addressed in two studies [48, 52] but no quantitative synthesis could be performed.

Minor peri-procedure complications were addressed in an RCT with 90 participants [45] and in five observational studies with 1390 patients [46, 48, 52, 56, 58]. Both studies reported a much lower incidence of hypocalcemia after hemithyroidectomy (relative risk *-* RR *-*, 0.12; 95% confidence interval *-* CI *-*, 0.06-0.25).

No study addressed the outcome of the cure for local signs and symptoms.

### 3.2 Comparison between Hemithyroidectomy and Thermoablation

This topic was addressed in one RCT with the use of MWA [46, 53] and four observational studies with the use of RFA [53], HIFU [54, 56], and MWA [55].

QoL was evaluated in two studies [45, 52] but no quantitative synthesis could be performed. The RCT [46] employed the questionnaire SF-36 and after a 6-month follow-up reported an improvement in general health, vitality, and mental health in the MWA group (N = 28) compared to the surgical group (N = 24). QoL scores were lower in the surgical group and superimposable in the MWA group when compared to the general population. In an observational study employing the HRQoL scale [53], the group treated with RFA achieved after 6 months a significantly higher improvement in general health, vitality, and mental health as compared to the surgical group (N = 108 for both groups).

The same RCT [46] did not observe any difference between the two groups for persistent local pain, acute hypocalcemia, and wound infection. An observational study of 101 patients [55] reported an increased risk of acute hypocalcemia in the surgical group as compared to MWA (RR, 13.61; 95% CI, 0.80-232.14). Three observational studies with 401 participants [54, 55, 57] showed an increased risk of wound infection or skin burn in the surgical group (RR, 4.54; 95% CI, 0.22-92.2).

### 3.3 Economic Evaluation

The mean cost per patient of the procedures under evaluation is € 4211,92 for hemithyroidectomy, € 5185,36 for total thyroidectomy, and € 1560,06 for thermoablation (Table **1**).

Table **2** Details the summary of evidence for the different domains evaluated by the panel.

### 3.4 Recommendations

Based on the reported analyses, the panel issued the following recommendations.

**Question 1.** What is the efficacy of hemithyroidectomy plus isthmectomy compared to total thyroidectomy for patients with a symptomatic benign thyroid nodule?

**Recommendation 1:** we suggest hemithyroidectomy plus isthmectomy, provided that clinically significant disease is not present in the contralateral thyroid lobe (weak recommendation, very low quality of evidence).

**Recommendation 2:** we suggest considering total thyroidectomy for patients with clinically significant disease in the contralateral thyroid lobe (weak recommendation, very low quality of evidence).

**Question 2.** What is the efficacy of hemithyroidectomy plus isthmectomy compared to ultrasound-guided ablative treatments for patients with a symptomatic benign thyroid nodule?

**Recommendation 3:** we suggest considering MIT as an alternative option to surgery for patients with a symptomatic, solid, benign, single, or dominant thyroid nodule (weak recommendation, very low quality of evidence).

### 3.5 Indications for Good Clinical Practice

The following statements reflect the opinions of the panel members about issues not addressed by studies directly comparing the different therapeutic options. These statements are complementary to the formal recommendations, are based on clinical experience, and are unanimously agreed upon by the panel. Thus, they are provided as an aid for good clinical practice.

1. The treatment choice for symptomatic benign thyroid nodules is based on neck US examination and FNA cytology report.
2. TSH-suppressing treatment with LT4 is not a routine option in euthyroid patients due to poor efficacy and potential side effects.
3. Radioiodine treatment, with or without rhTSH priming, is not an appropriate option due to modest volume reduction, slow symptom decrease, potential side effects, and risk of late hypothyroidism.
4. PEI should be considered as the first-line option for symptomatic benign, single or dominant, nodules which are completely or nearly completely cystic.
5. In patients with comorbidities, increased surgical risk, or refusal of surgery, US-guided TA procedures are the appropriate approach to symptomatic, benign thyroid nodules.
6. US-guided ablative procedures (PEI or TA, performed with different techniques) are safely performed in a day-hospital setting, unless patients’ clinical conditions warrant hospitalization.

### 3.6 Guideline Update

This systematic review will be updated with the use of the same search strings, three years from the date of the GL final approval. The ERT and the GL panel will assess the availability of new clinical data that could modify the overall quality of evidence and the risk/benefit ratio and, consequently, the formulation of the recommendations and their strength.

The GL panel will also consider updating, adding, or removing clinical questions or outcomes of interest and their relative relevance. In case of changes in clinical questions and/or critical outcomes, the process of evidence review and development of the recommendation will be performed again.

## 4 DISCUSSION

Hemithyroidectomy is a well-known and standardized procedure, while TA for thyroid nodules was introduced recently. TA is a less standardized treatment modality, is performed with different devices and techniques, and requires a specific operator’s training.

The key problem in the cost determination of MIT is the unavailability of consistent data about the various methodologies used for TA and the value of NHS reimbursement in the different Italian regions. Thus, a preliminary survey on this issue was performed on clinicians with specific expertise in MIT who are routinely involved in the management of thyroid nodular disease.

The direct cost of the surgical procedure results in the main expense for both hemithyroidectomy (72.45% of total) and total thyroidectomy (72.24%), while is only 57.58% for TA. It is worth noting that the cost item for health operators should be interpreted as a “cost-opportunity”. Italian NHS operators, indeed, are paid regardless of their specific activities.

The implementation of this GL [59] should not increase the costs generated by the management of thyroid nodules, though a conclusive assessment needs the evaluation of real practice data.

Only a quarter of thyroidectomies are now performed because of malignancy, as demonstrated by two large series in France [7] and Germany [8]. The average yearly number of thyroidectomies (summing up the total and partial operations) performed in Italy in the last 20 years was 40,000, but only a quarter of them was due to malignancy [60]. These data were retrieved from the hospital discharge forms, information that could be biased by a few drawbacks:

➢ Inconstant accuracy in the compilation.

➢ Occasional lack of histology report.

➢ Missing information about laboratory data, number and size of the nodules, US, and scintigraphic characteristics of the lesion.

➢ Procedures performed in private structures are not reported.

Based on their own clinical experience, panel members esteem that at least 30% of patients operated on for benign thyroid disease are affected by uni- or multi-nodular goiter with a dominant nodule that causes local pressure symptoms. Accordingly, a range of 8,000-10,000 patients per year could be suitable for TA. During the next three years, after the publication of this GL, the number of ablative treatments will not reach this potential estimate due to the insufficient availability of centers with skilled operators in our country. Thus, TA treatments will range from 3000 up to 9000/year, as a consequence of increased treatment accessibility.

According to cost estimates reported in this GL, the mean saving for each TA treatment performed in lieu of hemithyroidectomy could be € 2651 per patient (that is, € 4211 for hemithyroidectomy minus € 1560 for ablation). Consequently, the estimated yearly saving for NHS might range from an initial amount of 7,953,000 to a maximum of € 23,859,000, if the NHS accessibility to TA procedures will be adequate to satisfy all the requests.

Reimbursement for hemithyroidectomy, as for any procedure performed in public hospitals, is not derived from the plain sum of reported expenses but is controlled by regulatory agencies. In this regard the reimbursement for hemithyroidectomy corresponds to ICD9-CM 06.2, 06.3, and 06.51 that produce the DRG 290, corresponding to a maximum reimbursement of € 3340 for thyroidectomy without complications. TA treatments for thyroid nodular diseases are presently not coded by NHS. In clinical practice, most hospitals use the code ICD9-CM 06.98 for this issue which corresponds to “other surgical procedure on the thyroid gland”. The treatment should be performed in day-surgery and is reimbursed with € 1373. By applying the NHS rules, the difference between the costs of the two procedures can be estimated in € 1967. Accordingly, the yearly saving for NHS might range from a minimum amount of 5,901,000 (for 3000 TA-treated patients) to a maximum of € 17,703,000 (if all suitable patients would be treated).

A few limits in the calculation of the expected costs should be considered.

First, the estimated costs may change due to the fluctuation of the price of the disposable material employed during both surgery and TA. Second, the 15% rate of recurrency after TA that was presumed for this analysis might be higher if this procedure will be performed in centers without specific expertise. Third, cost analysis considered the same follow-up length for surgery and TA. This might imply an underestimation of costs associated with TA because it is presumable that the long-term follow-up after hemithyroidectomy might be less intensive. Fourth, the costs of possible replacement treatments after surgery and of its monitoring might change in the future. Finally, the cost for the operators during surgery should be increased because the reported calculation does not consider the loss of time between surgical procedures, such as re-setting of the operating room, preparation of the operators, and patients weaning from anesthesia.

## CONCLUSION

In conclusion, the available data suggest that the implementation of this GL recommendations will result in the progressive reduction of surgical procedures for benign thyroid nodular disease, with a decreased number of admissions to surgical departments for non-malignant conditions and more rapid access to patients with thyroid cancer. Importantly, a reduction of indirect costs due to long-term replacement therapy and the management of surgical complications may also be speculated.

Additionally, an improvement in the management of thyroid nodular disease and thyroid patients’ QoL may be achieved based on the indications for good clinical practice provided by the experts’ panel.

## Figures and Tables

**Table 1:** Comparison of the costs of the procedures.

**Procedure**	**Hemithyroidectomy**	**Total Thyroidectomy**	**Thermoablation**
**Before Treatment**			
**-**	€ 281.80	€ 281.80	€ 369.25
**Periprocedural**			
Drugs	€ 12.08	€ 11.49	€ 1.94
Materials	€ 149.97	€ 199.54	€ 661.98
Operators	€ 184.65	€ 232.47	€ 40.64
Operating room	€ 1356.98	€ 1685.28	-
Hospital stay	€ 1348.00	€ 1617.60	€ 193.78
Sub-total	**€ 3051.77**	**€ 3746.39**	**€ 898.34**
**Follow-up**			
Standard course	€ 105.34	€ 133.03	€ 91.11
Course with acute complications*	€ 7.98	€ 9.06	€ 7.93
Course with chronic complications°	€ 9.07	€ 10.40	€ 9.66
Sub-total	**€ 49.79**	**€ 152.50**	**€ 108.70**
**Loss of Patient’s Productivity**			
**§**	€ 755.97	€ 1004.68	€ 183.77
Gross total	**€ 4211.92**	**€ 5185.36**	**€ 1560.06**

**Note:** *Acute complications were estimated to involve 3.5% of patients.

°Chronic complications were estimated to involve 3.5% of patients.

§Caregivers were estimated to be involved in the assistance of 2.5% of patients undergoing hemithyroidectomy, 5% of patients undergoing total thyroidectomy, and 10% of patients undergoing thermoablation. Days required for recovery after the procedure were estimated, as a mean, 8.50, 11.22, and 1.82, respectively.

**Table 2:** Summary of evidence.

-	**Hemithyroidectomy *vs*. Thyroidectomy**	**Hemithyroidectomy *vs*. Thermoablation**
Desirable effects	Large	Moderate
Undesirable effects	Small	Moderate
Quality of evidence	Very low	Very low
Values	Probably large uncertainty or variability	Probably large uncertainty or variability
Balance of effects	Favours hemithyroidectomy	Probably favours thermoablation
Required resources	Moderate savings	Moderately higher costs
Quality of evidence for required resources	Moderate	Moderate
Cost-efficacy ratio	Probably favours hemithyroidectomy	Unknown
Equity	Unknown	Unknown
Acceptability	Probably yes	Probably no
Feasibility	Probably yes	Probably yes

## LIST OF ABBREVIATIONS

ABC: Activity Based Costing
AME: Associazione Medici Endocrinologi (Italian Association of Clinical Endocrinologists)
CI: Confidence Interval
CINHAL: Cumulative Index to Nursing and Allied Health Literature
DRG: Diagnosis Related Group
DTC: Differentiated Thyroid Carcinoma
EtD: Evidence to Decision
FNA: Fine-Needle Aspiration Biopsy
GL: Guideline
GRADE: Grading of Recommendations Assessment, Development and Evaluation
HIFU: High Intensity Focused Ultrasound
HRQoL: Health Related QoL
LT4: Levo-Thyroxine
LTA: Laser Ablation
MESH: Medical Subject Headings
MIT: Image-Guided Minimally Invasive Treatments
MWA: Microwave Ablation
NHS: National Health Service
OR: Odds Ratio
PEI: Percutaneous Ethanol Injection
PICO: Population, Intervention, Comparison, Outcome
QoL: Quality of Life
RCT: Randomized Controlled Trial
RFA: Radiofrequency Ablation
RR: Relative Risk
SF-36: The Short Form Health Survey
TA: Thermal Ablation Procedure
TIRADS: Thyroid Imaging Reporting and Data System
TSH: Thyrotropin
US: Ultrasonography
WMD: Weighted Mean Difference
