# Supplementary material for: Italian Guidelines for the Management of Non-Functioning Benign and Locally Symptomatic Thyroid Nodules
Source: Endocr Metab Immune Disord Drug Targets. 2023 Apr 19;23(6):876–85. doi: 10.2174/1871530323666230201104112 (PMC10245801; doi:10.2174/1871530323666230201104112)
Supplement: Supplementary file 1 — Supplementary material is available on the publisher’s website along with the published article. [file EMIDDT-23-876_SD1.pdf]

Supplementary Material

Italian Guidelines for the Management of Non-Functioning Benign and Locally Symptomatic Thyroid Nodules

Enrico Papini[1], Anna Crescenzi[2], Annamaria D'Amore[3], Maurilio Deandrea[4], Anna De Benedictis[5], Andrea Frasoldati[6], Roberto Garberoglio[7], Rinaldo Guglielmi[1], Celestino Pio Lombardi[3], Giovanni Mauri[8], Rosa Elisa Miceli[9], Soraya Puglisi[10], Teresa Rago[11], Domenico Salvatore[12], Vincenzo Triggiani[13], Dominique Van Doorne[14], Zuzana Mitrova[15], Rosella Saulle[15], Simona Vecchi[15], Michele Basile[16], Alessandro Scoppola[17], Agostino Paoletta[18], Agnese Persichetti[19], Irene Samperi[20], Renato Cozzi[21], Franco Grimaldi[22], Marco Boniardi[23]; Angelo Camaioni[24]; Rossella Elisei[11]; Edoardo Guastamacchia[13]; Giulio Nati[25]; Tommaso Novo[26]; Massimo Salvatori[27]; Stefano Spiezia[28]; Gianfranco Vallone[29]; Michele Zini[6]; Roberto Attanasio[30,*]

[1]Department of Endocrine and Metabolic Diseases, Ospedale Regina Apostolorum, Albano Laziale, Rome, Italy; [2]Department of Endocrine Organs and Neuromuscolar Pathology, Università Campus Bio-Medico di Roma, Rome, Italy; [3]Endocrine Surgery Division, Agostino Gemelli School of Medicine, University Foundation Polyclinic, Rome, Italy, [4]Endocrinology and Center for Thyroid Diseases, Ospedale Mauriziano "Umberto I", Turin, Italy; [5]Quality Management - Clinical Direction, Fondazione Policlinico Universitario Campus Bio-Medico, Rome, Italy; [6]Struttura Complessa di Endocrinologia, Arcispedale S. Maria Nuova, IRCCS, Reggio Emilia, Italy; [7]Freelancer at Thyroid Multidisciplinary Center at Humanitas Cellin , Turin, Italy; [8]Interventional Radiology, IRCCS European Institute of Oncology, Milan, Italy; [9]Private practice, Rome, Italy; [10]Department of Clinical and Biological Sciences, Internal Medicine, AOU San Luigi di Orbassano, University of Turin, Turin, Italy; [11]Department of Clinical and Experimental Medicine, University of Pisa, Pisa, Italy; [12]Department of Public Health, University Federico II, Naples, Italy; [13]Interdisciplinary Department of Medicine-Section of Internal Medicine, Geriatrics, Endocrinology and Rare Diseases, University of Bari, Bari, Italy; [14]Associazione Medici Endocrinologi, relationship with Patients' Associations, Rome Italy; [15]Department of Epidemiology, Lazio Region Health Service, Rome, Italy; [16]High School of Economy and Management of Health Systems, Catholic University of Sacred Heart, Rome, Italy; [17]Department of Endocrinology, Ospedale Santo Spirito, Rome, Italy; [18]Department of Endocrinology, ULSS6 Euganea, Padova, Italy; [19]Department of Firefighters, Public Rescue and Civil Defense, Ministry of Interior, Rome, Italy; [20]Department of Endocrinology, ASL Novara, Novara, Italy; [21]President of Associazione Medici Endocrinologi, Milan, Italy; [22]Past-president of Associazione Medici Endocrinologi, Udine, Italy; [23]General Oncologic and Mini-invasive Surgery Department, ASST Grande Ospedale Metropolitano Niguarda, Milan, Italy; [24]Otolaryngology Department, San Giovanni-Addolorata Hospital, Rome, Italy; [25]ASL Roma, Rome, Italy; [26]Department of Endocrinology, Santa Maria Nuova Hospital, Turin, Italy; [27]Nuclear Medicine Unit, Fondazione Policlinico Universitario A. Gemelli IRCCS and Department of Radiological and Hematological Sciences, Catholic University of Sacred Heart, Rome Italy; [28]Department of Endocrine and Ultrasound-Guided Surgery, Ospedale del Mare, Naples, Italy; [29]Department of Radiology, Federico II University Hospital, Naples Italy; [30]AME Scientific Committee, Milan, Italy

APPENDIX 1:

Guideline Development Team

Chair: Enrico Papini (endocrinologist, Endocrinology & Metabolism Department, Ospedale Regina Apostolorum, Albano Laziale).

Panel Members:

- Anna Crescenzi (pathologist, Pathology Department, Fondazione Policlinico Universitario Campus Biomedico, Rome)
- Annamaria D'Amore and Celestino Pio Lombardi (endocrine surgeons, Endocrine Surgery, Fondazione Policlinico Universitario Agostino Gemelli - IRCCS, Catholic University of Sacred Heart, Rome)
- Maurilio Deandrea (endocrinologist, Endocrinology and Thyroid Diseases Center, Ospedale Mauriziano "Umberto I", Turin)
- Anna De Benedictis (nurse, Direzione Clinica, Quality Manager, Fondazione Policlinico Universitario Campus Biomedico, Rome)
- Andrea Frasoldati (endocrinologist, Endocrinology Unit, Arcispedale S. Maria Nuova IRCCS, ASL Reggio Emilia)
- Roberto Garberoglio (nuclear physician and radiologist, private practice, Turin)
- Rinaldo Guglielmi (endocrinologist, Endocrinology & Metabolism Department, Ospedale Regina Apostolorum, Albano Laziale)
- Giovanni Mauri (interventional radiologist, Interventional Radiology Unit, European Institute of Oncology, Milan)
- Rosa Elisa Miceli (psychologist and psychotherapist, private practice, Rome)
- Paola Polano (lawyer, Patients' Association ATTA Lazio, Rome)
- Soraya Puglisi (endocrinologist, Department of Clinical and Biological Sciences, 1st Internal Medicine, AOU San Luigi di Orbassano, University of Turin)
- Teresa Rago (endocrinologist, Endocrinology Unit, Department of Clinical and Experimental Medicine, University of Pisa)
- Vincenzo Triggiani (endocrinologist, Endocrinology and Metabolic Diseases, Department of Medicine, University "Aldo Moro", Bari)
- Dominique Van Doorne (endocrinologist, AME supervisor for relationship with Patients' Associations, Rome)

EVIDENCE REVIEW TEAM

- Laura Amato, Fabio Cruciani, Zuzana Mitrova, Rosella Saulle, Simona Vecchi (Department of Epidemiology, Regional Health Service - ASL Roma1, Regione Lazio)
- Michele Basile (High School of Economy and Management of Health Systems, Catholic University of Sacred Heart, Rome)

EXTERNAL REVIEWERS

- Marco Boniardi (endocrine surgeon, Endocrine Surgery Unit – ASST Grande Ospedale Metropolitano Niguarda – Milan)
- Angelo Camaioni (endocrine surgeon, Ear Nose & Throat Department, Ospedale S. Giovanni - Addolorata, Rome)
- Rossella Elisei (endocrinologist, Endocrinology Unit, Department of Clinical and Experimental Medicine, University of Pisa)
- Edoardo Guastamacchia (endocrinologist, Endocrinology and Metabolic Diseases, Department of Medicine, University “Aldo Moro”, Bari)
- Giulio Nati (endocrinologist, general practitioner, Rome)
- Tommaso Novo (nurse, Turin)
- Massimo Salvatori (nuclear physician, Department of Imaging, Oncologic Radiotherapy and Hematology, Institute of Nuclear Medicine, Fondazione Policlinico Universitario Agostino Gemelli - IRCCS, Catholic University of Sacred Heart, Rome)
- Stefano Spiezia (endocrine surgeon, Endocrine and Ultrasound Guided Surgery, Ospedale del Mare, Naples)
- Gianfranco Vallone (radiologist, AOU Federico II, Naples)
- Michele Zini (endocrinologist, Endocrinology Unit, Arcispedale S. Maria Nuova IRCCS, ASL Reggio Emilia)

AME GUIDELINE TEAM

- Alessandro Scoppola, team coordinator (endocrinologist, Endocrinology Unit, Ospedale Santo Spirito, Rome)
- Roberto Attanasio (endocrinologist, AME Scientific Committee, Milan)
- Lino Furlani (endocrinologist, Endocrinology Unit, Ospedal Negrar, VR)
- Agostino Paoletta (endocrinologist, ULSS6 Euganea Endocrinology, Padova)
- Enrico Papini (endocrinologist, Endocrinology & Metabolism Department, Ospedale Regina Apostolorum, Albano Laziale, Rome)
- Agnese Persichetti: Ministry of Interior - Department of Firefighters, Public Rescue and Civil Defense, Rome
- Irene Samperi (endocrinologist, Endocrinology Unit, ASL Novara)

APPENDIX 2: POPULATION-INTERVENTION-COMPARISON-OUTCOME (PICO)

Clinical question: What is the efficacy of hemithyroidectomy plus thyroid isthmus resection vs. total thyroidectomy vs. ablative procedures, vs. other non-invasive treatments, vs. no treatment for patients with benign symptomatic thyroid nodules?

Population: adult subjects (aged ≥18 years) diagnosed with benign symptomatic thyroid nodules.

Intervention: hemithyroidectomy plus thyroid isthmus resection.

COMPARISONS:

a) total thyroidectomy.
b) imaging-guided ablative procedures:

- ethanol injection.
- laser thermoablation.

- radiofrequency thermoablation.
- high intensity focused ultrasound thermoablation.
- microwave thermoablation.

c) other non-invasive treatments:

- TSH-suppressive or semi-suppressive L-T4 treatment.
- iodine supplementation.
- food integrators.
- radiometabolic treatment (^{131}I).

d) no intervention (clinical observation).

OUTCOMES

1. cure of local signs and symptoms:
    - pressure (cervical constriction, dysphagia, cough, dysphony, respiratory impairment, pain).
    - esthetic (local lump).
2. Permanent cure (risk of re-intervention).
3. Major peri-procedure complications (death, local bleeding, injury to vital cervical structures).
4. Minor peri-procedure complications (persisting local pain, acute hypocalcemia, wound infection, dehiscence or burn, lymphatic duct lesion).
5. Side effects (due to local or general anesthesia, peri-procedure pain, absence from work).
6. Permanent complications (mortality at 30 days, tracheostomy for bilateral recurrent nerve palsy, permanent dysphonia, hypothyroidism requiring replacement treatment, permanent hypoparathyroidism, permanent cosmetic damage, functional injury to cervical nervous structures).
7. Quality of life (relational and work discomfort, perception of one's own appearance, self-esteem, need for therapeutic adherence, local and general symptom anxiety).

Design of searched studies: systematic reviews and metanalysis of RCTs or individual RCTs. If unavailable, observational studies with a control group were searched.

Exclusion criteria: pregnant women, patients with hyperfunctioning nodules, Hashimoto's thyroiditis, or Graves' disease; studies comparing different techniques for surgery (open vs. endoscopic, etc.).

APPENDIX 3 – SEARCH STRATEGY

Database: The Cochrane Library

Date of search: Issue 6, 2020

#1 MeSH descriptor: [Thyroid Nodule] explode all trees

#2 MeSH descriptor: [Goiter, Nodular] this term only

#3 (thyroid* NEAR (nod* or incidentalom* or goiter)):ti,ab,kw

#4 ((goiter* or goitre*) near (nodul* or multinodul* or multi nodul* or nontoxic or non toxic)):ti,ab,kw

#5 #1 OR #2 OR #3 OR #4

#6 MeSH descriptor: [Thyroidectomy] explode all trees

#7 thyroidectom*:ti,ab

#8 surgery:ti,ab,kw

#9 #6 OR #7 OR #8

#10#5 AND #9

DATABASE: OVID MEDLINE(R)

Date of search: 1946 to June 08, 2020

1. exp Thyroid Nodule/

2. exp Goiter, Nodular/
3. (thyroid* adj6 (nod* or incidentalom* or goiter)).tw,ot.
4. ((goiter* or goitre*) adj6 (nodul* or multinodul* or multi nodul* or nontoxic or non toxic)).tw.
5. ((thyroid adj3 (neoplasm or tumor)) and benign).tw.
6. Thyroidectomy/
7. thyroidectom*.tw.
8. 1 or 2 or 3 or 4 or 5
9. 6 or 7
10. 8 and 9

Filter for systematic reviews

1. meta-analysis/ or systematic review/ or meta-analysis as topic/ or "meta analysis (topic)"/ or "systematic review (topic)"/ or exp technology assessment, biomedical/
2. ((systematic* adj3 (review* or overview*)) or (methodologic* adj3 (review* or overview*))).ti,ab,kf,kw.
3. ((quantitative adj3 (review* or overview* or synthes*)) or (research adj3 (integrati* or overview*))).ti,ab,kf,kw.
4. ((integrative adj3 (review* or overview*)) or (collaborative adj3 (review* or overview*)) or (pool* adj3 analy*)).ti,ab,kf,kw.
5. (data synthes* or data extraction* or data abstraction*).ti,ab,kf,kw.
6. (handsearch* or hand search*).ti,ab,kf,kw.
7. (handsearch* or hand search*).ti,ab,kf,kw.
8. (meta regression* or metaregression*).ti,ab,kf,kw.
9. (meta-analy* or metaanaly* or systematic review* or biomedical technology assessment* or bio-medical technology assessment*).mp,hw.
10. (medline, cochrane, PubMed, medlars, embase, or cinahl).ti,ab,hw.
11. (cochrane or (health adj2 technology assessment) or evidence report).jw.
12. (comparative adj3 (efficacy or effectiveness)).ti,ab,kf,kw.
13. (outcomes research or relative effectiveness).ti,ab,kf,kw.
14. ((indirect or indirect treatment or mixed-treatment) adj comparison*).ti,ab,kf,kw.
15. 11 or 12 or 13 or 14 or 15 or 16 or 17 or 18 or 19 or 20 or 21 or 22 or 23 or 24
16. 10 and 25
17. ((clinical adj3 pathways) or (practice adj3 parameter) or (practice adj3 parameters)).ti,ab,kw. or algorithms/ or care pathway.ti,ab,kw. or care pathways.ti,ab,kw. or clinical protocols/ or Consensus/ or Consensus Development Conference.pt. or Consensus Development Conference, NIH.pt. or Consensus Development Conferences as Topic/ or Consensus Development Conferences, NIH as Topic/ or critical pathway/ or guidance.ti,ab. or guideline*.ti. or guidelines as topic/ or practice guidelines as topic/ or Health Planning Guidelines/ or practice guideline/
18. 10 and 27
19. 26 or 28

Filter for RCT

1. randomized controlled trial.pt.
2. controlled clinical trial.pt.
3. random*.ab.
4. placebo.ab.
5. clinical trials as topic.sh.
6. random allocation.sh.
7. trial.ti.
8. 10 or 11 or 12 or 13 or 14 or 15 or 16
9. exp animals/ not humans.sh.
10. 37 not 38
11. 29 AND 10
12. 39 AND 10

DATABASE: EMBASE

Date of search: 1974 to 2020 June 08

1. Thyroid Nodule/
2. Nodular goiter/
3. (thyroid* adj6 (nod* or incidentalom* or goiter)).tw,ot.
4. ((goiter* or goitre*) adj6 (nodul* or multinodul* or multi nodul* or nontoxic or non toxic)).tw.
5. ((thyroid adj3 (neoplasm or tumor)) and benign).tw.
6. Thyroidectomy/
7. thyroidectom*.tw.
8. 1 or 2 or 3 or 4 or 5
9. 6 or 7
10. 8 and 9

Filter for systematic reviews

1. "systematic review"/ or meta analysis/
2. "meta analysis (topic)"/
3. "systematic review (topic)"/
4. biomedical technology assessment/
5. ((systematic* adj3 (review* or overview*)) or (methodologic* adj3 (review* or overview*))).ti,ab.
6. ((quantitative adj3 (review* or overview* or synthes*)) or (research adj3 (integrati* or overview*))).ti,ab.
7. ((integrative adj3 (review* or overview*)) or (collaborative adj3 (review* or overview*)) or (pool* adj3 analy*)).ti,ab.
8. (data synthes* or data extraction* or data abstraction*).ti,ab.
9. (handsearch* or hand search*).ti,ab.
10. (mantel haenszel or peto or der simonian or dersimonian or fixed effect* or latin square*).ti,ab.
11. (met analy* or metanaly* or technology assessment* or HTA or HTAs or technology overview* or technology appraisal*).ti,ab.
12. (meta regression* or metaregression*).ti,ab.
13. (meta-analy* or metaanaly* or systematic review* or biomedical technology assessment* or bio-medical technology assessment*).mp,hw.
14. (medline, Cochrane, PubMed, medlars, embase, or cinahl).ti,ab.
15. (cochrane or (health adj2 technology assessment) or evidence report).jw.
16. (comparative adj3 (efficacy or effectiveness)).ti,ab.
17. (outcomes research or relative effectiveness).ti,ab.
18. ((indirect or indirect treatment or mixed-treatment) adj comparison*).ti,ab.
19. 11 or 12 or 13 or 14 or 15 or 16 or 17 or 18 or 19 or 20 or 21 or 22 or 23 or 24 or 25 or 26 or 27 or 28
20. 10 and 29
21. exp clinical pathway/
22. exp clinical protocol/
23. exp consensus/
24. exp consensus development conference/
25. exp consensus development conferences as topic/
26. critical pathways/
27. guidelines as topic/
28. exp practice guideline/
29. practice guidelines as topic/
30. health planning guidelines/
31. (position statement* or policy statement* or practice parameter* or best practice*).ti.
32. (standards or guideline or guidelines).ti,kw.
33. ((practice or treatment* or clinical) adj guideline*).ab.
34. consensus*.ti,kw.
35. ((critical or clinical or practice) adj2 (path or paths or pathway or pathways or protocol*)).ti,ab,kw.

36. recommendat*.ti.
37. (care adj2 (standard or path or paths or pathway or pathways or map or maps or plan or plans)).ti,ab,kw.
38. 31 or 32 or 33 or 34 or 35 or 36 or 37 or 38 or 39 or 40 or 41 or 42 or 43 or 44 or 45 or 46 or 47
39. 10 and 48
40. 30 or 49
41. 50 AND 10

Filter for RCT

1. Clinical-Trial/ or Randomized-Controlled-Trial/ or Randomization/ or Single-Blind-Procedure/ or Double-Blind-Procedure/ or Cross-over-Procedure/ or Prospective-Study/ or Placebo/
2. (((clinical or control or controlled) adj (study or trial)) or ((single or double or triple) adj (blind$3 or mask$3)) or (random$ adj (assign$ or allocat$ or group or grouped or patients or study or trial or distribut$)) or (crossover adj (design or study or trial)) or placebo or placebos).ti,ab.
3. 52 or 53
4. 10 and 54

Database: Web of Science

#1 TS= clinical trial* OR TS=research design OR TS=comparative stud* OR TS=evaluation stud* OR TS=controlled trial* OR TS=follow-up stud* OR TS=prospective stud* OR TS=random* OR TS=placebo* OR TS=(single blind*) OR TS=(double blind*)

#2 TS=(thyroi* NEAR/6 (nod* or incidentalom* or goiter))

#3 TS=((goiter* or goitre*) NEAR/6 (nodul* or multinodul* or nontoxic or "non toxic"))

#4 #2 OR #3

#5 TI=thyroidectom*

#6 #1 AND #4 AND #5

DATABASE: CINAHL (EBSCO)

Date of search: 1974 to 2020 June 16

Filter for systematic reviews

S1 ((MH "Random Assignment") or (MH "Random Sample+") or (MH "Crossover Design") or (MH "Clinical Trials+") or (MH "Comparative Studies") or (MH "Control (Research)+") or (MH "Control Group") or (MH "Factorial Design") or (MH "Quasi-Experimental Studies+") or (MH "Placebos") or (MH "Meta Analysis") or (MH "Sample Size") or (MH "Research, Nursing") or (MH "Research Question") or (MH "Research Methodology+") or (MH "Evaluation Research+") or (MH "Concurrent Prospective Studies") or (MH "Prospective Studies") or (MH "Nursing Practice, Research-Based") or (MH "Solomon Four-Group Design") or (MH "One-Shot Case Study") or (MH "Pretest-Posttest Design+") or (MH "Static Group Comparison") or (MH "Study Design") or (MH "Clinical Research+")) or (clinical nursing research or random* or cross?over or placebo* or control* or factorial or sham* or meta?analy* or systematic review* or blind* or mask* or trial*)

S2 (MH "Thyroid Nodule")

S3 TI ((thyroi* N6 (nod* or incidentalom* or goiter))) OR AB ((thyroi* N6 (nod* or incidentalom* or goiter)))

S4 TI (((goiter* or goitre*) N6 (nodul* or multinodul* or multi nodul* or nontoxic or non toxic))) OR AB (((goiter* or goitre*) N6 (nodul* or multinodul* or multi nodul* or nontoxic or non toxic)))

S5 S2 OR S3 OR S4

S6 (MM "Thyroidectomy")

S7 TI Thyroidectom* OR AB Thyroidectom*

S8 S6 OR S7

S9 S5 AND S8

S10 S1 AND S5 AND S8

DATABASE: Web of Science

Date of search: 1997 to October 13, 2020

Indexes=SCI-EXPANDED, SSCI, A&HCI, CPCI-S, CPCI-SSH, ESCI Timespan=All years

10 #9 AND #3

9 #8 OR #7 OR #6 OR #5 OR #4

8 TS=(lobectomy NEAR/2 (thyroid* or goiter* or goitre*))

7 TS="Thyroid Lobectomy"
6 TS=Isthmectomy
5 TS=hemithyroidectomy
4 TS=(partial NEAR/2 thyroidectomy*)
3 #2 OR #1
2 TS=((goiter* or goitre*) NEAR/6 (nodul* or multinodul* or nontoxic or "non toxic"))
1 TS=(thyroid* NEAR/6 (nod* or incidentalom* or goiter))

APPENDIX 4 – STUDY SELECTION

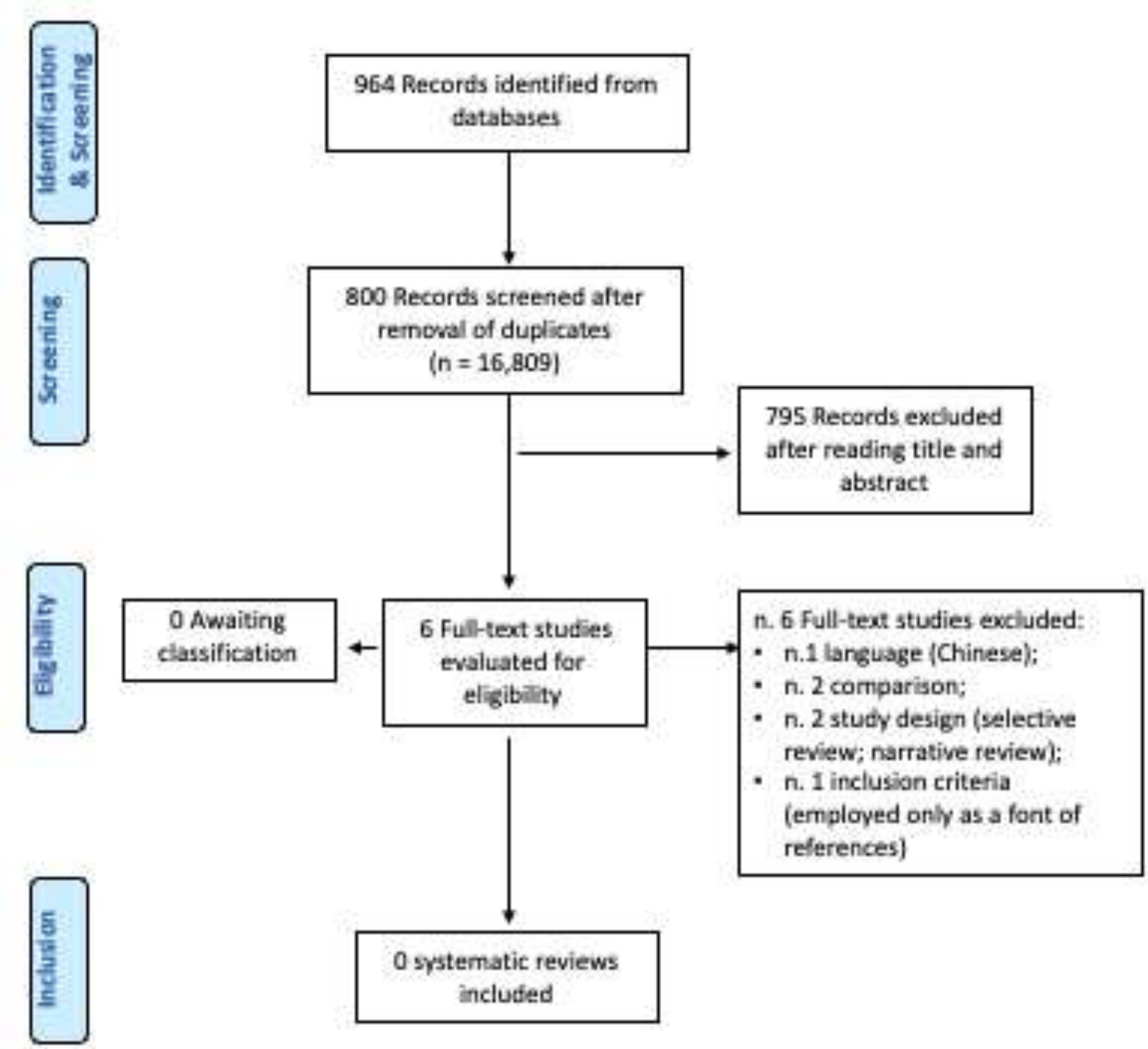

Fig. (1). Selection for systematic reviews.

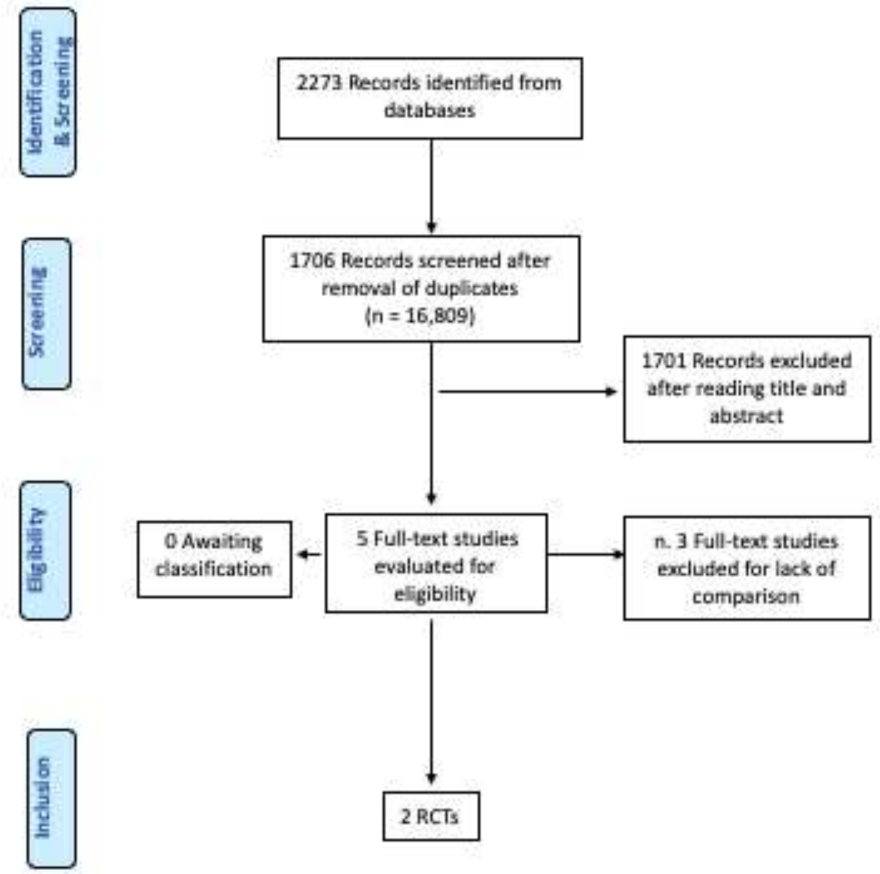

Fig. (2). Selection for RCTs

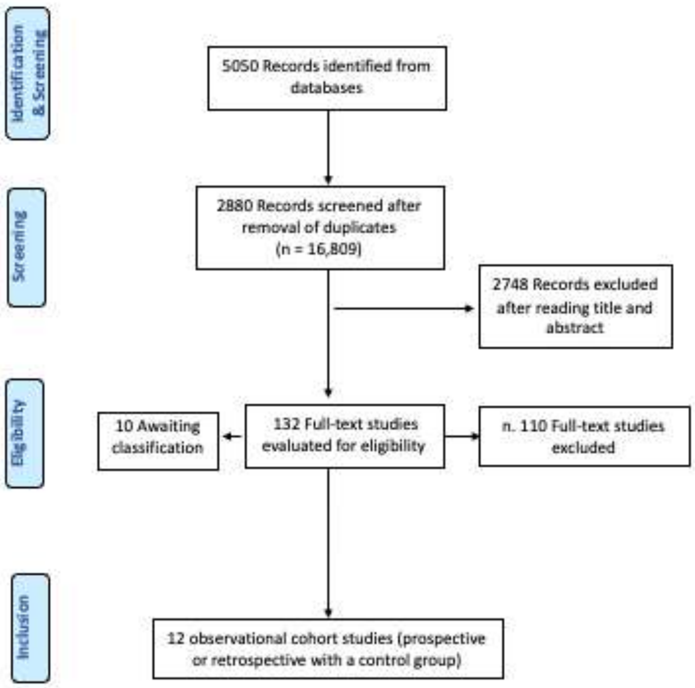

Fig. (3). Selection for observational cohort studies (retrospective/prospective) with a control group.

APPENDIX 5 —EVALUATION OF METHODOLOGIC QUALITY OF INCLUDED STUDIES

RCT (Cochrane Tool)

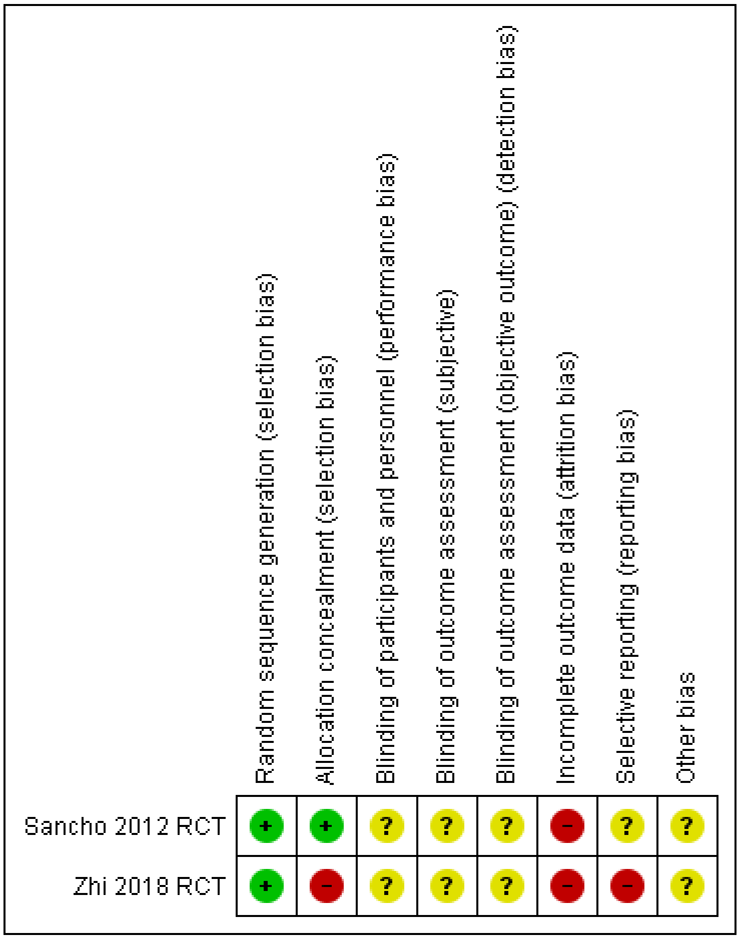

OBSERVATIONAL STUDIES (NEWCASTLE-OTTAWA SCALE)

-	Selection (max 4 stars)	Comparability (max 2 stars)	Outcomes (max 3 stars)	Total
Bauer 2013	****	-	***	7
Cossu 1999	**	-	**	4
Dong 2020	**	-	**	4
Lang 2019	****	-	**	6
Lang 2017	****	-	***	7
Mourad 2001	****	-	***	7
Scerrino 2001	**	-	**	4
Simsek Celik 2010	****	-	***	7
Tabriz 2020	*	*	**	4
Vaiman 2008	***	-	***	6
Vaiman 2010	***	-	***	6
Yue 2016	****	**	**	8
